# Improvement in muscular strength and aerobic capacities in elderly people occurs independently of physical training type or exercise model

**DOI:** 10.6061/clinics/2019/e833

**Published:** 2019-06-20

**Authors:** Mari L Sbardelotto, Rochelle R Costa, Karine A Malysz, Giulia S Pedroso, Bárbara C Pereira, Helen R Sorato, Paulo C L Silveira, Renata T Nesi, Antônio J Grande, Ricardo A Pinho

**Affiliations:** I Universidade Integrada do Alto Uruguai e das Missões, Erechim, RS, BR; II Laboratorio de Fisiologia e Bioquimica do Exercicio, Programa de Pos-Graduacao em Ciencias da Saude, Unidade de Ciencias da Saude, Universidade do Extremo Sul Catarinense, Criciuma, SC, BR; III Laboratorio de Bioquimica do Exercicio em Saude, Faculdade de Medicina, Programa de Pos-Graduacao em Ciencias da Saude, Pontificia Universidade Catolica do Parana (PUCPR), Curitiba, PR, BR; IV Laboratorio de Evidencias baseadas em Saude, Programa de Pos-Graduacao de Educacao em Saude, Faculdade de Medicina, Universidade Estadual do Mato Grosso do Sul, Campo Grande, MS, BR

**Keywords:** Aging, Physical Exercise, Physical Fitness, Lipid Profile

## Abstract

**OBJECTIVES:**

Progressive decline of physiological processes with aging is normal. Aging is also associated with decreased functional capacity and onset of many diseases. This study evaluated the changes in physical fitness (PF), body composition (BC), and lipid profile (LP) in elderly men completing different training protocols.

**METHODS:**

Fifty-five men (age 60-80 years) were randomized into the following groups: without training, aerobic training on dry land, combined training on dry land, and combined training in water. Training was conducted for 8 weeks, and PF, LP, and BC were assessed at the beginning and end of the intervention.

**RESULTS:**

Significant improvements were observed in all parameters; however, combined programs on land or in water were more effective at improving strength and aerobic fitness. Combined exercise produced greater effects on BC and LP and some muscle fitness parameters; however, improvements in muscular and aerobic capacities occurred independently of exercise type or model.

**CONCLUSION:**

These results indicate that the effects of training occur regardless of training type or model, and are directly associated with training periodization, adherence, and regularity.

## INTRODUCTION

Aging is a process inherent to human life that involves the progressive decline in all physiological processes and that, therefore, becomes an important concern because of the consequent reduced functional capacity and impaired quality of life 1. A natural occurrence in the aging process is sarcopenia, which involves the degenerative loss of muscle mass, strength, and function 2. For instance, the skeletal muscle mass in humans decreases by almost 50% in old age, and muscle strength is reduced by 15% per decade starting from the age of 50 years, with the rate of reduction potentially reaching 30% per decade 3. Although sarcopenia is not a disease, its severity is associated with various age-related diseases, and the condition can be harmful to the health of elderly persons, in particular because it is directly related to the onset or worsening of chronic diseases 4.

Evidence accumulated in recent years indicates that adopting a healthy lifestyle, which includes a healthy diet, physical exercise, stress management, and social interaction, can substantially minimize the deleterious effects of aging 5. Physical exercise has been indicated as an important intervention in treatments aimed at minimizing the deleterious effects of aging, particularly sarcopenia. According to the guidelines published by the World Health Organization 6 about the importance of physical activity in elderly people, exercise is a cost-effective method of preventing the decline in functional capacity in elderly people and can help prevent and manage certain chronic diseases and conditions.

Biological aging is associated with reduced muscle mass, strength, and cardiorespiratory fitness, resulting in an impaired ability to perform daily activities 7. Because of this, the type of exercise is a determining factor in improving the physical fitness of elderly people. Several recommendations about physical exercise for elderly people have been made in different consensus statements. These consensus statements generally recommend 300 min of moderate training or, for additional health benefits, 150 min of vigorous-intensity exercise per week 8. The American College of Sports Medicine and the American Heart Association 9 recommend 30 min of moderate-intensity aerobic exercise 5 times per week or 20 min of vigorous exercise 3 times per week. Both recommendations suggest performing resistance exercises 2 or more days per week. These recommendations, although important, are nonspecific. Given this issue, recent studies have proposed more effective exercise protocols for aged individuals 10-12.

Bann et al. 13 suggested that a combination of strength and aerobic exercises for elderly populations seems to be the most effective strategy for improving both neuromuscular and cardiorespiratory function and, consequently, maintaining functional capacity during aging. This combination seems important because each of these exercise types has different effects on the organs. Adaptations to strength training promote improvements in muscle strength and hypertrophy, neural adaptations, increases in motor unit recruitment, increases in maximum motor unit firing rate, and increases in neuronal excitability 14,15. On the other hand, aerobic resistance training induces central and peripheral adaptations that increase VO_2 max_ and the ability of skeletal muscle to generate energy through oxidative metabolism without a concurrent increase in muscle strength or hypertrophy 16. In addition, osteoarticular and muscular changes such as time-induced balance changes should be considered when choosing the exercise environment and the type of exercise. Although the literature has not clarified this issue, water training seems to be a good alternative for reducing the gravitational impact on the body 17 and providing positive health benefits 18. However, the results comparing the effects of exercise carried out in different environments are still inconclusive.

The mechanisms behind the favorable effects of exercise are not yet clear because of the lack of clarity about the optimal exercise type, intensity, and frequency. Thus, the objective of this study was to evaluate changes in the levels of physical fitness and the lipid profiles of elderly men performing different physical training protocols.

## METHODS

### Experimental design and participants

This parallel randomized controlled trial was conducted at a physical fitness complex in Rio Grande do Sul, Brazil. Participants were recruited by means of radio programs, and randomization was subsequently performed. The eligibility criteria for participation in this study were as follows: no use of drugs or medications capable of inducing myopathy, no physical limitations or clinical diseases that might compromise the execution of the exercise, and no regular exercise for at least 6 months immediately prior to the study. All of the selected subjects were instructed not to perform any type of physical training outside of the study protocol.

### Randomization and blinding

**Figure 1:**
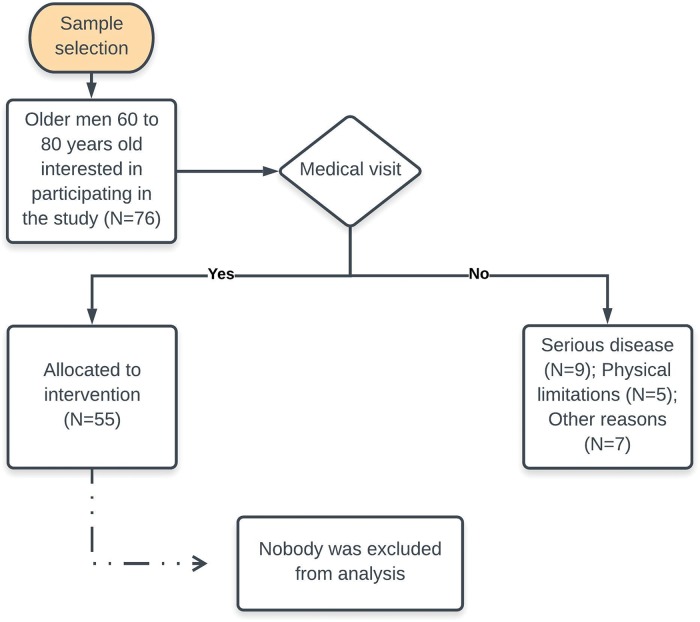
Fifty-five participants were randomly divided into four groups: control (Ctr, n=14), aerobic training on dry land (ATdl, n=12), combined training on dry land (CTdl, n=12), and combined training in water (CTw, n=17). The sample size was not calculated. We chose to widely publicize the research on a local radio station and to conduct the research with as many participants as possible. The participants received explanations regarding the purpose and risks of the protocol and signed informed consent forms. The participant characteristics are listed in Table 1. This study was approved by the Research Ethics Committee of the Universidade do Extremo Sul Catarinense (protocol number 119477/72012).

A total of 76 elderly people expressed interest in participating in this study. Of them, 21 did not meet the inclusion criteria. The flowchart of participant inclusion is shown in Figure 1. After the initial screening for participants fulfilling the inclusion criteria, the researchers wrote numbers 1-4 (indicating each group) on small pieces of paper, which were folded and placed in an opaque plastic bag. In exceptional cases, some participants were allocated to a specific group according to medical advice, regardless of the draw. The baseline measurements of elderly participants who underwent the different physical training protocols are presented in Table 1.

### Interventions

The training protocols were performed over a period of 8 weeks, with 3 exercise sessions per week. Each 1-h session was divided into 3 parts: 5-min warm-up exercise; 50-min specific training (aerobic, strength, or combined); and a final 5-10-min phase involving resting the back, whole-body stretching, exercises for joint mobility and specific muscle groups as determined during training, or some form of recreation to ensure program adherence. All training protocols were performed in a linear progression of volume, intensity, and recovery, and arranged in mesocycles.

### Aerobic training on dry land (ATdl)

A treadmill was used for this training model. The heart rate was monitored and maintained between 60% and 80% of the maximum heart rate during the main training phase (Table 2).

### Combined training on dry land (CTdl)

Aerobic exercise was performed as in the ATdl group on dry land. The strength exercises were conducted in a circuit with exercises involving large muscle groups (pec deck, basic abdominal exercises, leg press, inverted peck deck, and extensor exercises) and small muscle groups (bicep, calf, tricep, and abductor/adductor exercises). The exercise sessions were performed in a series of 12 repetitions for each exercise with an intensity of 60-70% of 1 repetition maximum (1 RM) and with 2 min of active recovery between the series (Table 3).

### Combined training in water (CTw)

The exercise sessions were performed in circuits involving the upper limbs (internal and external rotation of the shoulders with simultaneous and individual arm exercises) and the lower limbs (alternate and simultaneous leg kicks). Aerobic exercises (various activities) were conducted at the end of the training session for 20 min with an intensity of 60-80% of the maximum heart rate (Table 4).

### Data collection

Data were collected at 2 specific time points. The first collection (baseline data) was performed 72 h before the first training session, and the second collection was performed 48 h after the last training session.

### Anthropometric parameters

For height measurements, we used a wall-mount stadiometer (WCS, Brazil). For body mass assessments, we used a mechanical scale with a capacity of up to 150 kg and a precision of 0.1 kg (Arja, Brazil). Measurements of the triceps, supra-iliac, and abdominal skin folds were acquired using a caliper with an accuracy of 1 mm (Mitutoyo/CESCORF, Brazil), whereas abdominal and hip circumferences were measured using a flexible tape measure with an accuracy of 1 mm (SANNY, Brazil).

### Neuromuscular fitness

Neuromuscular fitness was assessed through a 1 RM test in which the heaviest load that could be lifted by the participant in a full repetition of the movement was determined 19. The 1 RM test focused on the knee extensors, horizontal shoulder extensors, and shoulder flexors, according to the protocol.

### Functional fitness of elderly people

The functional capacity of the participants was assessed using the senior fitness test according to the method of Rikli and Jones 20; method test consists of 5 tests: strength and endurance of the lower limbs (rise from and sit on a chair for 1 min), strength and endurance of the upper limbs (forearm flexion for 1 min), flexibility of the lower limbs (sit and reach), flexibility of the upper limbs (reaching behind the back), and walking speed (sit, walk 2.44 m, and sit again).

### Cardiorespiratory fitness

Cardiorespiratory fitness was assessed using the Rockport mile test 21, in which the participants had to walk 1609 m at a steady pace as fast as possible. To assess cardiorespiratory fitness, we used walking time, sex, age, body weight, and heart rate as measured immediately after the walk.

### Lipid profile

Blood levels of triglycerides (TGs), total cholesterol (TC), low-density lipoprotein cholesterol (LDL-c), very-low-density lipoprotein cholesterol (VLDL-c), and high-density lipoprotein cholesterol (HDL-c) were measured using specific kits from Roche and analyzed using a Reflotron Plus (Roche Diagnostics, Switzerland). The relationships among these measures were assessed by calculating the TC/HDL-c and LDL-c/HDL-c ratios.

### Statistical analysis

The data are expressed as the means and standard errors. For the analysis, we used the generalized estimating equations, which enable the analysis of continuous outcomes even when variables do not show a normal distribution or sphericity 22, with a post hoc Bonferroni correction. Values of *p*<0.05 were considered significant. Statistical processing was performed by a trained researcher who was blinded to the participants' groups using SPSS software (Statistical Package for Social Sciences for Mac, version 22.0).

## RESULTS

### Lipid profile

A lipid profile analysis (Table 5) revealed that the different models of physical training resulted in significant absolute and relative improvements in lipid levels compared to the levels prior to the intervention. The decrease in TC, LDL-c, and the TC/HDL-c and LDL-C/HDL-c ratios in all training models were significant compared to those in the control group. Similar improvements were observed in the HDL-c fraction which had a significant increase after training across all training models and a significant decrease in the control group. Moreover, a significant decrease in the TG and VLDL-c levels was observed in the ATdl and CTw groups compared with the level in the control group. In addition, the change in percentage of the variables was similar in all the groups studied.

### Functional fitness

**Figure 2:**
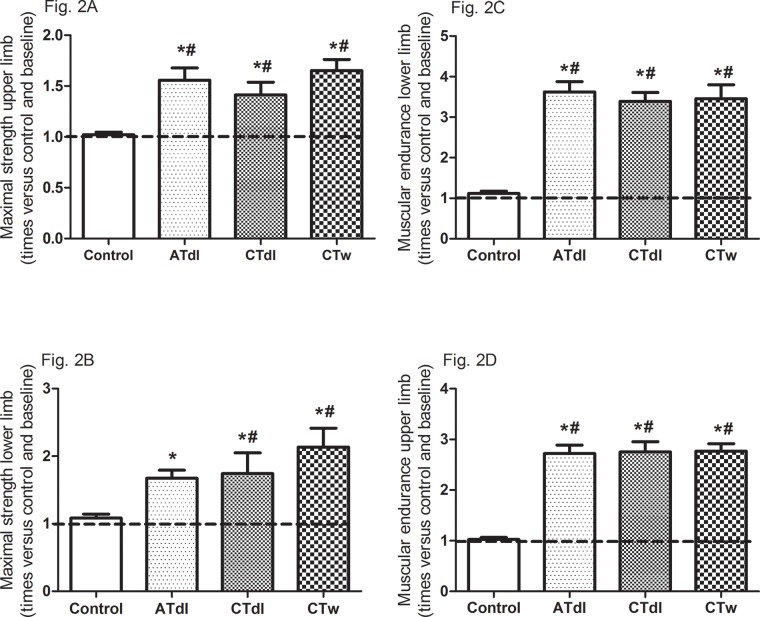
**(A-D).** Effects of different training protocols on muscle strength (**2A**, upper limb, peck deck; **2B**, lower limb, extension; **2C**, upper limb, forearm flexing; **2D**, lower limb, getting up and sitting down) of elderly people after 2 months of intervention with aerobic training on dry land (ATdl), combined training on dry land (CTdl), or combined training in water (CTw) three times a week for 60 min per session. The dotted line represents the baseline values of each group. The generalized estimating equations with Bonferroni post hoc correction were used to detect intragroup significant differences. *Baseline *versus* posttraining and between groups, #training groups *versus* control in the posttraining period with a significance level of *p*<0.05.

**Figure 3:**
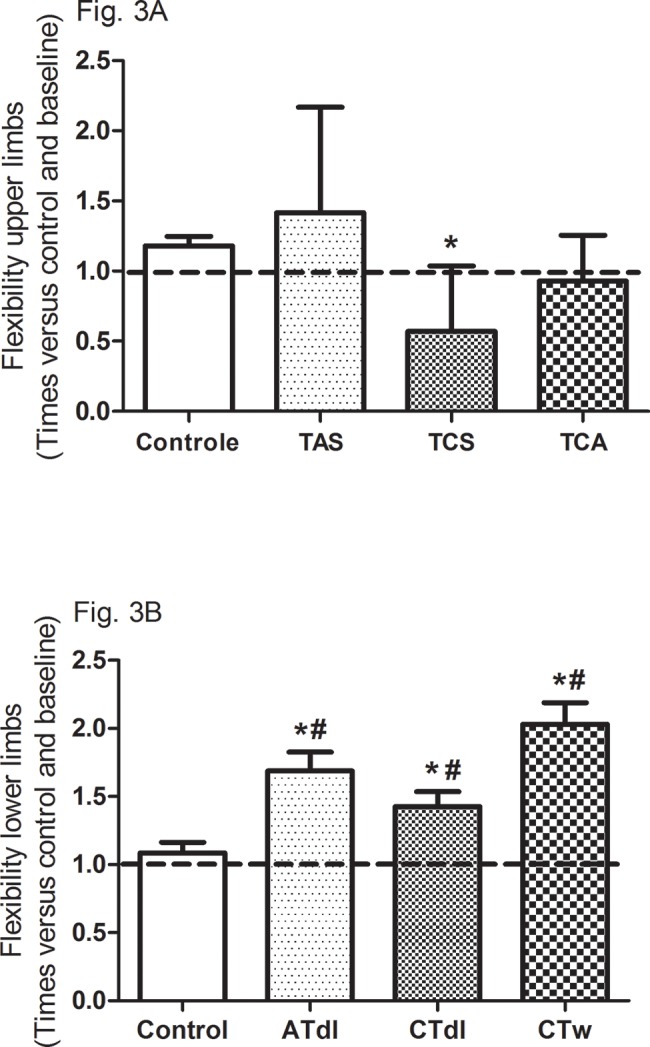
Effects of different training protocols on flexibility (**3A**, upper limb; **3B**, lower limb) of elderly people after 2 months of intervention with aerobic training on dry land (ATdl), combined training in dry land (CTdl), or combined training in water (CTw) three times a week for 60 min per session. The dotted line represents the baseline values of each group. The generalized estimating equations with Bonferroni post hoc correction were used to detect the intragroup significant differences. *Baseline *versus* posttraining and between groups, #training groups *versus* control in the posttraining period with a significance level of *p*< 0.05.

**Figure 4:**
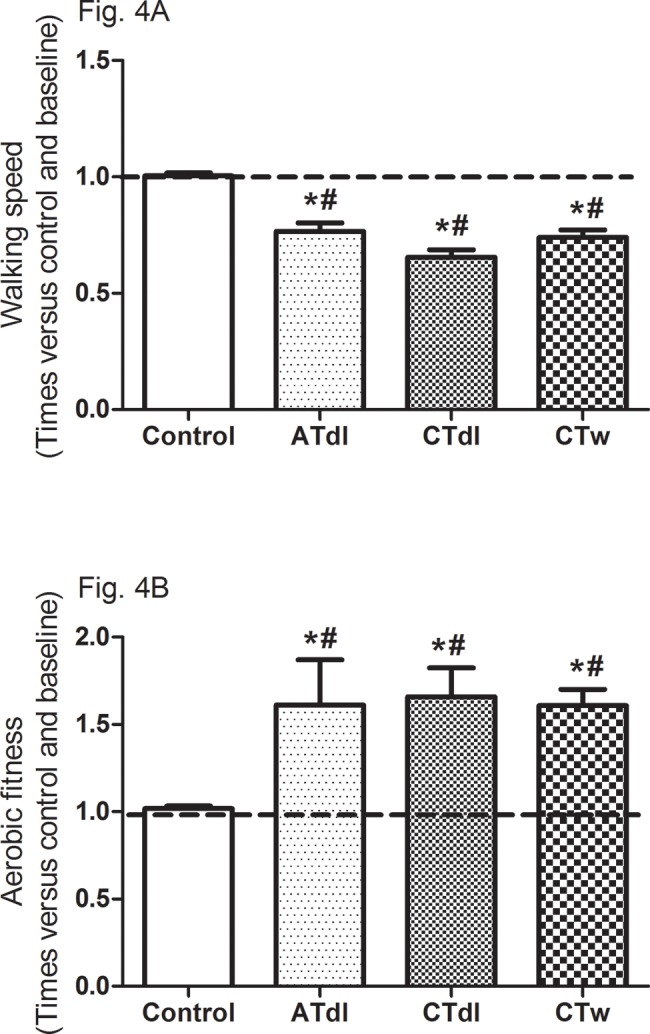
Effects of different training programs on walking speed and aerobic fitness (**4A** and **4B**, respectively) of elderly individuals after 2 months of intervention with aerobic training on dry land (ATdl), combined training on dry land (CTdl), or combined training in water (CTw) three times a week for 60 min per session. The dotted line represents the baseline values of each group. The generalized estimating equations with Bonferroni post hoc correction were used to detect the intragroup significant differences. *Baseline *versus* posttraining and between groups, #training groups *versus* control in the posttraining period with a significance level of *p*<0.05.

All training protocols (ATdl, CTdl, and CTw) resulted in significant improvements in all variables indicating physical fitness in elderly men. The maximum muscle strength (Figure 2A and B) and muscular endurance were assessed (Figure 2C and D), and the results showed that the 3 training protocols resulted in significant improvements compared to the baseline values and compared to the values in the control group. Figure 3 (A and B) shows the effects of training on flexibility. Participants in the CTdl group had decreased upper-limb flexibility after training compared to their flexibility at baseline, whereas lower-limb flexibility was significantly increased in participants in the 3 training groups compared to their flexibility at baseline. Only the ATdl and CTw groups showed significant improvements in flexibility compared to the flexibility of the control group. The results of walking speed (Figure 4A) showed that the training protocols significantly decreased the time required by the elderly participants to walk a given distance compared to the baseline time and to the control group time. In addition, aerobic fitness (Figure 4B) improved significantly across the 3 training groups compared to the baseline values and to the control group.

## DISCUSSION

Aging induces neuromuscular disorders related to spinal motor neuron degeneration and decreased muscle fiber number and size, which, in turn, led to losses in muscle performance that compromise an individual's independence 23. In addition, longitudinal studies have shown an age-related decline in aerobic fitness 11,24,25. However, Vincent et al. 26 suggested that, regardless of the physical training model, exercise is an effective intervention for functional decline, sarcopenia, and frailty. All types of exercise improve functional capacity and mitigate risk factors for sarcopenia, including inflammation, oxidative stress, and insulin resistance. Deslandes et al. 27 reviewed the evidence accumulated in the last 20 years on the efficacy of exercise (both aerobic and strength exercises) in preventing and treating diseases and in decreasing the deleterious effects of aging 27. However, although longitudinal studies report that exercise regularity is more important than exercise type and that the effects of exercise on some parameters depend on the training model, we considered a crossover verification of the effects of different types of physical training important. Combined training that promotes the development of strength and aerobic fitness in the same session seems to be an effective strategy for maintaining or improving functional capacity in elderly people. However, most studies have recommended aerobic training models. Hence, we devised an experimental protocol in which both strength and aerobic training were included in the same training session in water or on dry land to investigate the effects of combined training on the physical fitness and lipid profiles of elderly men.

The effects of exercise on blood lipid levels in elderly people have been widely studied 28 because, with aging, the blood lipid composition undergoes significant changes associated with changes in body composition or the onset of diseases associated with old age. In this study, we observed a significant decrease in the levels of TC, LDL-c, TC/HDL-c, and LDL-C/HDL-c and a significant increase in HDL-c levels in 3 training models. Moreover, a significant decrease was observed in the TG and VLDL-c levels of the ATdl and CTw groups compared to the levels of the control group. According to Carrick-Ranson et al. 29, the positive effects of different training models may be associated with the training frequency. The authors suggested that 4 or more weekly exercise sessions performed throughout life promote significant changes in lipid profile levels regardless of the training model. Similar results were also observed in recent studies of an experimental rodent model 30 and of humans 31. Mann et al. 32 evaluated the effects of 3 training models (aerobic, strength, and combined) on the lipid profile. They observed a strong dose-response relationship between lipid profile and energy expenditure regardless of the training model. Therefore, the benefits of exercise are independent of the specific features and characteristics of the training model. However, regular exercise seems to be the most important factor associated with an improvement in lipid profile.

Regular exercise can lead to improved physical fitness and, subsequently, a greater ability to perform tasks throughout an individual's lifetime. The results of this study showed that the training programs positively influenced the functional fitness parameters (strength, muscular endurance, flexibility, walking speed/agility, and aerobic fitness) of the elderly participants. The baseline upper- and lower-limb strength and flexibility and walking speed/agility were classified as weak, according to Rikli and Jones 20; however, after the intervention period, those parameters were significantly improved in all training groups compared to the measurements at baseline. In particular, muscular strength, which is usually related to muscle function loss and to a potential decrease in the ability to synthesize proteins with age, is considered the main factor in the development of sarcopenia according to Deutz et al. 33.

In addition to muscular fitness, aerobic fitness is an important parameter of physical fitness in elderly people, and the results of this study showed significant improvement in aerobic fitness after the different training programs. Supporting these findings, Chapman et al. 34 observed similar results of cardiovascular fitness in aged individuals after a 12-week aerobic training intervention. Erickson et al. 35 evaluated the association between physical activity and cardiorespiratory fitness and the volume of gray matter in the brain of older adults and suggested that higher levels of cardiorespiratory fitness are routinely associated with a greater volume of gray matter in the prefrontal cortex and hippocampus. Aging is associated with decreased neural integrity, whereas cardiovascular fitness, which can potentially improve the quality of life, delay cognitive decline, and prolong functional fitness in elderly people by ensuring functional independence, is a factor that might mitigate the neural decline in neural integrity related to age 36.

Taken together, the results of this study showed that it is possible to infer that the muscle functional capacity and the aerobic capacity of elderly people are improved by exercise regardless of the training type or model; however, the combined training programs on land or in water are more effective than individual training programs in promoting changes in strength and aerobic fitness. The reasons for these results are unclear, especially with respect to aerobic fitness, as no strength component was included in the ATdl protocol. In addition to the aerobic component, combined training can ensure greater motivation for the practice of exercise in elderly people because it involves more types of exercises. With respect to combined training in water, greater body control and more safety measures are needed by elderly people in performing the exercises. In addition, water provides a low-impact environment and minimizes the risk of injury or undue stress on the joints. Overall, all these factors tend to make the water training program more enjoyable and could increase the adherence to training, which results in better improvements in the muscular fitness of elderly people.

## CONCLUSION

Our findings suggest that improvements in muscle functional capacity and aerobic capacity may result from exercise programs regardless of the training type or model used and that these results are directly associated with training periodization, adherence, and regularity. However, the combined programs on land or in water, aimed at developing variable maximum strength and aerobic fitness in the same session, seem to be more effective at improving strength and aerobic fitness than an isolated aerobic training model.

### Limitations

This study has some limitations due to the specificity of the population. We were not able to fully control the use of medications or to stratify the participants into smaller age groups. Although it is known that dual-energy X-ray absorptiometry is a reference assessment technique for muscle mass evaluation and has been considered the gold standard method for sarcopenia diagnosis, we did not measure body composition with this method owing to technical limitations at the time of data collection.

## AUTHOR CONTRIBUTIONS

Sbardelotto ML designed and performed the study and wrote the manuscript. Costa RR and Malysz KA participated in the data collection and conducted the training programs. Pedroso GS, Pereira BC and Sorato HR participated in the data collection. Silveira PCL, Nesi RT and Grande AJ contributed to the design and manuscript writing. Pinho RA supervised, designed and helped writing the manuscript.

## Figures and Tables

**Table 1:** Baseline measures of elderly individuals submitted to different physical training protocols.

			Training					
Variables	Control		ATdl		CTdl		CTw	
	Mean±sd	Min/Max	Mean±sd	Min/Max	Mean±sd	Min/Max	Mean±sd	Min/Max
AGE (years)	67.5±6.52	60/78	67.17±4.41	60/75	67.42±6.73	60/78	68.06±6.35	60/79
HEIGHT (cm)	173.03±5.8	164.5/183	172.76±5.21	164/181	171.46±5.52	157/178	173.59±6.27	158.5/182
BM (kg)	74.18±4.33	65.71/82.66	86.27±3.33	79.63/92.80	72.57±2.56	67.56/77.59	83.23±2.95	77.45/89.02
FAT (%)	21.41±2.62	16.29/26.55	23.78±1.51	20.82/26.75	20.56±2.00	16.63/24.48	24.86±1.18	22.55/27.17
LM (kg)	57.01±1.88	53.32/60.69	65.35±2.00	61.43/69.27	56.91±1.73	53.51/60.31	61.90±1.55	58.87/64.94
BMI (kg.m^-2^)	25.18 ±1.72	21.80/28.57	28.93±1.04	26.90/30.97	24.48±0.75	23.02/25.95	27.75±0.91	25.96/29.53
HR_INITIAL REST_	69.09±4.81	60/75	67.75±6.05	60/78	68.33±5.14	60/75	67.76±4.32	61/75
HR_ FINAL REST_	69.64±4.18	61/76	64.75±6.22	55/75	65.08±4.62	59/72	63.59±3.67	58/70

Age, body fat percentage (%), lean mass (LM), and body mass index (BMI) were determined. Groups: control (Ctr, n=14), aerobic training on dry land (ATdl, n=12), combined training on dry land (CTdl, n=12), and combined training in water (CTw, n=17). All data are presented as the mean and standard deviation (sd).

**Table 2:** Aerobic training program.

Mesocycle	Microcycle (Weeks)	Sessions per mesocycle	Series per session	Time of exercise in the series	Intensity (% Fcmáx)	Time of active recovery between series	Total time
1	1 - 2	6	5	5 min	70%	1 min	29 min
2	3 - 5	9	4	8 min	75%	1 min	35 min
3	6 - 8	9	3	15 min	80%	2 min	47 min

The aerobic training program on dry land (ATdl). The training was performed on a treadmill. Treadmill speed was adjusted individually for each participant and the heart rate was controlled using a Polar monitor.

**Table 3:** Combined training program on dry land.

				Strength exercise				Aerobic exercise		
Mesocycle	Microcycle (Weeks)	Sessions	Series	Repetition	Intensity % 1RM	Active Recovery (min)	Time of exercise	Intensity (% Fcmáx)	Active recovery	Total
1	1 - 2	6	3	12 x	60%	2 min	5 min	70%	1 min	36 min
2	3 - 5	9	3	10 x	70%	2 min	5 min	75%	1 min	45 min
3	6 - 8	9	3	08 x	80%	3 min	5 min	80%	1 min	68 min

The combined training program on dry land (aerobic/strength, CTdl). The training program was performed in a gym and focused on the knee extensors and horizontal shoulder extensors and flexors. The exercise sessions were organized in the form of sequential circuit by segments and five types of exercise involving large muscle groups: peck deck, basic abdominals, leg press, inverted peck deck, and extension; and four exercises involving small muscle groups: biceps, calves, triceps, and abductors/adductors. Movement speed was adjusted individually to maintain the heart rate within the target zone during training. The heart rate was controlled using a Polar monitor.

**Table 4:** Combined training program in water.

				Strength exercise				Aerobic exercise		
Mesocyce	Microcycle (Weeks)	Sessions	Series	Time in the series	Execution speed	Active recovery	Time of exercise	Intensity (% Fcmáx)	Active recovery	Total
1	1 - 2	6	2	30 sec	maximum	1 min	10 min	70%	1 min	44 min
2	3 - 5	9	3	20 sec	maximum	1 min	15 min	75%	1 min	49 min
3	6 - 8	9	4	15 sec	maximum	1 min	20 min	80%	1 min	59 min

Combined training program in water (aerobic/strength, CTw). The training program was performed in a swimming pool (15 × 25 meters) and focused on the knee extensors and horizontal shoulder extensors and flexors. The exercise sessions were organized in the form of a sequential circuit by segments and five types of exercise involving large muscle groups: peck deck, basic abdominals, leg press, inverted peck deck, and extension; and four exercises involving small muscle groups: biceps, calves, triceps, and abductors/adductors. Movement speed was adjusted individually to maintain the heart rate within the target zone during training. The heart rate was controlled using a Polar monitor.

**Table 5:** Lipid profile measures of elderly individuals in physical training groups.

Training								
Variables	Control		ATdl		CTdl		CTw	
	Baseline	Post-training	Baseline	Post-training	Baseline	Post-training	Baseline	Post-training
TC	198.21±10.54	214.21 ±11.26*	223.34 ±10.41	203 ±10.42*	219.71 ±16.96	179.67 ±11.99*	224.06 ±11.47	191.06 ±11.58*
Difference (%)	+8% (*p*=0.001)		-9% (*p*=0.000)		-18% (*p*=0.005)		-14% (*p*=0.000)	
HDL-c	46.85 ±5.51	40.71 ±4.48*	41.33 ±4.04	54.25 ±4.75*	42.92±4.76	56.67 ±5.09*	32.94 ±1.69	45.76 ±1.48*
Difference (%)	-15% (*p=0.008)		+41% (*p=0.000)		+32% (*p=0.000)		+39% (*p=0.000)	
LDL-c	123.71±12.26	137.86±12.51*	145.67 ±10.43	119.83 ±11.02*	147.17 ±18.3	97.17 ±13.61*	142.65 ±11.45	98.53 ±12.84*
Difference (%)	+11% (*p=0.008)		-18% (*p=0.000)		-34% (*p=0.003)		-30% (*p=0.000)	
VLDL-c	26.93 ±3.21	38.8±3.43*	36.33 ±4.75	29.92 ±3.39*	29.5 ±3.35	26.17 ±2.72^#^	61.12 ±8.78	36 ±3.94*
Difference (%)	+44% (*p=0.000)		-17% (*p=0.001)		-11% (#*p*=024)		-40% (*p=0.001)	
TC/HDL-c	4.76 ±0.49	10.15±2.4*	6.05 ±0.62	3.87 ±0.35*	5.97 ±0.78	3.5 ±0.39*#	7.01 ±0.55	3.99 ±0.32*
Difference (%)	+113% (*p=0.037)		-36% (*p=0.000)		-41% (*p=0.000; #*p*=0.038)		-44% (*p=0.000)	
LDL-c/HDL-c	3.09 ±0.45	4.04±0.6*	4.04 ±0.53	2.25 ±0.31*	4.15 ±0.69	1.97 ±0.36*#	4.59 ±0.48	2.17 ±0.32*#
Difference (%)	+30% (*p=0.023)		-44% (*p=0.023)		-52% (*p=0.000; #*p*=0.023)		-52% (*p=0.000; #*p*=0.043)	
TG	138.36±15.68	216.15 ±32.92*	181.5±23.76	144.5 ±16.83*	148.17 ±16.81	128.58 ±13.22	242.76 ±28.77	170.06 ±12.97*
Difference (%)	+56% (*p=0.000)		-20% (*p=0.001)		-13%		-30% (*p=0.001)	

Means and standard error of the mean of the lipid profile variables: total cholesterol (TC), high density lipoproteins linked to HDL-c cholesterol, low density lipoproteins linked to LDL-c cholesterol, very-low-density lipoproteins linked to VLDL-c cholesterol, TC/HDL-C ratio, LDL-c/HDL-c ratio, and TG triglycerides at baseline and after 2 months of physical training three times a week for 60 minutes per session. Analysis of the generalized estimating equations with the Bonferroni post hoc test was used to detect the intragroup statistical differences. *Baseline *versus* post-training and between groups, #trained groups *versus* control in the post-training period considering a significance index of *p*<0.05.
